# Inhibition of the ubiquitin-proteasome system reduces the abundance of pyruvate dehydrogenase kinase 1 in cultured myotubes

**DOI:** 10.1007/s10974-024-09679-3

**Published:** 2024-07-31

**Authors:** Blaž Kociper, Nives Škorja Milić, Ivana Ogrizek, Katarina Miš, Sergej Pirkmajer

**Affiliations:** Institute of Pathophysiology, Faculty of Medicine, University of Ljubljana, Zaloška 4, Ljubljana, 1000 Slovenia

**Keywords:** Pyruvate dehydrogenase kinase 1 (PDK1), Myotubes, Hypoxia-inducible factor-1α (HIF-1α), MG132, PYR-41, Chloroquine

## Abstract

Pyruvate dehydrogenase kinase (PDK), which phosphorylates the pyruvate dehydrogenase complex, regulates glucose metabolism in skeletal muscle. PDK1, an isozyme whose expression is controlled by hypoxia-inducible factor-1α (HIF-1α), is thought to play a role in muscle adaptation to hypoxia. While transcriptional upregulation of PDK1 by HIF-1α is well characterised, mechanisms controlling proteolysis of PDK1 in skeletal muscle have not been thoroughly investigated. Proteasome inhibitor MG132 paradoxically reduced the abundance of PDK1 in human cancer cells and rat L6 myotubes, suggesting that MG132 might direct PDK1 towards autophagic degradation. The objectives of our current study were to determine (1) whether MG132 suppresses PDK1 levels in primary human myotubes, (2) whether chloroquine, an inhibitor of autophagy, prevents MG132-induced suppression of PDK1 in L6 myotubes, and (3) whether PYR-41, an inhibitor of ubiquitination, suppresses PDK1 in L6 myotubes. Using qPCR and/or immunoblotting, we found that despite markedly upregulating HIF-1α protein, MG132 did not alter the PDK1 expression in cultured primary human myotubes, while it suppressed both PDK1 mRNA and protein in L6 myotubes. The PDK1 levels in L6 myotubes were suppressed also during co-treatment with chloroquine and MG132. PYR-41 markedly increased the abundance of HIF-1α in primary human and L6 myotubes, while reducing the abundance of PDK1. In L6 myotubes treated with PYR-41, chloroquine increased the abundance of the epidermal growth factor receptor, but did not prevent the suppression of PDK1. Collectively, our results suggest that cultured myotubes degrade PDK1 via a pathway that cannot be inhibited by MG132, PYR-41, and/or chloroquine.

**Supplementary Information:**

The online version contains supplementary material available at 10.1007/s10974-024-09679-3.

## Introduction

Pyruvate dehydrogenase kinase (PDK) controls glucose metabolism in skeletal muscle, a major site of glucose oxidation (Brooks and Mercier 1994; Kelley et al. 1988), by phosphorylating the α1 subunit of pyruvate dehydrogenase (PDHE1α) in the pyruvate dehydrogenase complex (Bowker-Kinley et al. 1998; Pilegaard et al. 2006; Rardin et al. 2009; Sugden and Holness 2003). PDK-induced phosphorylation of PDHE1α inhibits the conversion of pyruvate to acetyl-CoA, thus suppressing glucose oxidation (Linn et al. 1969; Sugden and Holness 2003). All four PDK isozymes (PDK1-4) are expressed in skeletal muscle, but PDK2 and PDK4 are markedly more abundant than PDK3 and PDK1 (Bowker-Kinley et al. 1998; Gudi et al. 1995; LeBlanc et al. 2004; Spriet et al. 2004). However, only PDK1 phosphorylates PDHE1α at all three inhibitory sites (Ser293 [site 1], Ser300 [site 2], and/or Ser232 [site 3]), including the PDK1-specific Ser232 (Korotchkina and Patel 2001). PDK1 has a higher specific activity and is less sensitive to inhibition by pyruvate or its synthetic analogue dichloroacetate (DCA) than PDK2 and PDK4 (Bowker-Kinley et al. 1998; Whitehouse and Randle 1973). Phosphorylation catalysed by PDK1, which incorporates more phosphate into PDHE1α than other isozymes, results in slower reactivation of pyruvate dehydrogenase activity (Kolobova et al. 2001). Despite relatively low expression of PDK1 (Bowker-Kinley et al. 1998; Gudi et al. 1995; LeBlanc et al. 2004; Spriet et al. 2004) and although PDK2 is normally responsible for the majority of PDK activity in muscle tissue (Klyuyeva et al. 2019), PDK1 can effectively compensate for the loss of PDK activity in PDK2 knock-out mice (Dunford et al. 2011). In addition, the PDK1-specific Ser232 is prominently phosphorylated in skeletal muscle (Rardin et al. 2009), suggesting a role for PDK1 in the regulation of the pyruvate dehydrogenase complex in skeletal muscle.

Expression of the PDK1 gene is upregulated by hypoxia-inducible factor-1α (HIF-1α), an important mechanism by which cancer cells switch from oxidative to anaerobic glucose metabolism (Kim et al. 2006; Papandreou et al. 2006). A similar mechanism appears to exist in skeletal muscle, as when rats and mice are exposed to hypoxia, HIF-1α and PDK1 are upregulated in skeletal muscle (De Palma et al. 2007; Le Moine et al. 2011). In mice exposed to 12% O_2_ for 24 h, upregulation of PDK1 protein was accompanied by an increase in phosphorylation of PDHE1α at the PDK1-specific Ser232 (Le Moine et al. 2011), emphasising the functional importance of PDK1 in hypoxic skeletal muscle. Since PDK1 protein levels were unchanged (in limb muscles) or reduced (in the diaphragm) in humans and mice exposed to prolonged or intermittent hypoxia (Gamboa and Andrade 2010; Le Moine et al. 2011; Martinez-Bello et al. 2011), PDK1 upregulation in response to acute hypoxia appears to occur transiently.

Gene expression is important for regulation of PDK function in response to hypoxia (Kim et al. 2006; Papandreou et al. 2006) and a range of other stimuli (Majer et al. 1998; Pilegaard et al. 2006; Wu et al. 1999). However, while PDK1 protein levels in skeletal muscle were higher in trained than untrained subjects, mRNA levels were similar in both groups (Gudiksen et al. 2018). In the hearts of diabetic rats, the PDK1 protein levels were also increased without the corresponding mRNA changes (Wu et al. 1998). In addition, the PDK inhibitor DCA decreased the PDK1 protein levels in rat L6 myotubes without affecting its mRNA levels (Skorja Milic et al. 2021). The lack of concordance between PDK1 mRNA and PDK1 protein levels indicates that both transcriptional and post-transcriptional mechanisms contribute to regulation of PDK1 in skeletal muscle.

In cancer cells, ubiquitination of PDK1 leads to its proteasomal degradation (Cao et al. 2021; Yoshino et al. 2016), suggesting that the ubiquitin-proteasome system may regulate PDK1 levels in skeletal muscle. However, in rat L6 myotubes MG132, a proteasome inhibitor (Tsubuki et al. 1996), reduced PDK1 protein levels despite a marked upregulation of HIF-1α (Skorja Milic et al. 2021). The first aim of the current study was to determine whether suppression of PDK1 by MG132 is conserved in primary human myotubes. Since inhibition of the proteasome by MG132 can direct proteins towards autophagic degradation (Wang et al. 2019), the second aim of the study was to determine whether chloroquine, an inhibitor of autophagy (Dong et al. 2019; Klionsky et al. 2016), prevents the reduction of PDK1 protein levels in L6 myotubes treated with MG132. It was also important to consider that MG132 may only have an indirect effect on PDK1 protein levels due to its suppressive effect on gene transcription (Bhat et al. 2009). The third aim of the study was therefore to investigate whether PYR-41, an inhibitor of ubiquitination (Yang et al. 2007), mimics the effects of MG132 on PDK1 in L6 myotubes.

## Methods

### Materials and reagents

The rat L6 skeletal muscle cell line was purchased from ATCC (Manassas, VA, USA). Cell culture flasks and plates were from TPP (Trasadingen, Switzerland) or Sarstedt (Nümbrecht, Germany). Advanced MEM, DMEM, MEMα, foetal bovine serum (FBS), Pen Strep (5000 U/mL penicillin and 5000 µg/mL streptomycin), Fungizone (250 µg/mL amphotericin B), Immobilon Crescendo Western HRP Substrate Millipore, High-Capacity cDNA Reverse Transcription Kit, MicroAmp optical 96-well reaction plates, MicroAmp optical adhesive sheets, TaqMan Universal Master Mix and TaqMan gene expression assays for human HIF-1α (Hs00153153_m1), PDK1 (Hs01561847_m1), PGK1 (4333765F), and cyclophilin (PPIA) (Hs99999904_m1) and rat *Pdk1* (Rn00587598_m1) and *Ppia* (Rn00690933) were from Thermo Fisher Scientific (Waltham, MA, USA). The 4–12% Criterion™ XT Bis-Tris polyacrylamide gels, XT MES electrophoresis buffer, and goat anti-rabbit IgG-horseradish peroxidase conjugate were from Bio-Rad (Hercules, CA, USA). The Amersham ECL Full-Range Rainbow Molecular Weight Marker was from GE Healthcare Life Sciences Cytiva (Marlborough, MA, USA). The polyvinylidene difluoride (PVDF) membrane was from Merck Millipore (Burlington, MA, USA). The primary antibodies are listed in Table 1. PYR-41, MG132, chloroquine, dichloroacetate (DCA), actinomycin D, and puromycin were from Sigma-Aldrich (St. Louis, MO, USA). The E.Z.N.A. HP Total RNA Isolation Kit was from Omega Bio-tek (Norcross, GA, USA). All other reagents were from Sigma-Aldrich unless otherwise stated.

**Table 1:** Primary and secondary antibodies used for immunoblotting. Abbreviations: CST: Cell Signalling Technology, O/N: overnight

		Primary Antibody					Secondary Antibody					
Target protein	kDa	Supplier	Cat. No.	Ab Host	Dilution	Time		Supplier	Cat. No.	Dilution	Time	
ACC	280	CST	3676	Rb	1:1000	O/N		Bio-Rad	1706515	1:20,000	5 min	
Caspase 3	17, 19, 35	CST	9662	Rb	1:1000	O/N		Bio-Rad	1706515	1:10,000	1 h	
EGFR	175	CST	2232	Rb	1:1000	O/N		Bio-Rad	1706515	1:10,000	1 h	
HIF-1α	120	CST	3434	Rb	1:1000	O/N		Bio-Rad	1706515	1:8,000	1 h	
p-PDHE1α (Ser293)	43	Abcam	ab92696	Rb	1:1000	O/N		Bio-Rad	1706515	1:50,000	1 h	
PDK1	47	CST	3820	Rb	1:1000	O/N		Bio-Rad	1706515	1:5,000	1 h	
STAT3	79, 86	CST	4904	Rb	1:1000	O/N		Bio-Rad	1706515	1:15,000	5 min	

### Primary human skeletal muscle cells

Ethical authorisation was granted by the Republic of Slovenia Medical Ethics Committee (KME: 71/05/12 and 0120–698/2017/4). The donors of muscle tissue signed a written informed consent. Primary human skeletal muscle cells were prepared and cultured as described (Jan et al. 2021; Pavlin et al. 2024; Pirkmajer et al. 2020; Vidovic et al. 2024). Briefly, human skeletal muscle cell cultures were obtained from satellite cells isolated from the *semitendinosus* muscle samples that were discarded as surgical waste during routine anterior cruciate ligament reconstruction. The muscle tissue was stripped of connective and adipose tissue, cut into small pieces and trypsinized at 37 °C for 45 min to release the muscle satellite cells. The isolated cells were grown in 100 mm Petri dishes in growth medium (Advanced MEM supplemented with 10% (v/v) FBS, 0.3% (v/v) Fungizone, 0.15% (v/v) Gentamicin, GlutaMax, and vitamins) at 37 °C and in humidified air with 5% (v/v) CO_2_. The purity of myoblast cultures was increased using the CD56 magnetic-activated cell sorting (MACS, Miltenyi Biotec) system as described (Jan et al. 2021; Mars et al. 2020; Pirkmajer et al. 2020). CD56, also known as the neural-cell adhesion-molecule (NCAM), was shown to be useful for the enrichment of myogenic cells (de Luna et al. 2006; Sinanan et al. 2004). Before confluence was reached, the primary human skeletal muscle cells were trypsinized and separated into myogenic (CD56+) and non-myogenic (CD56-) fractions (Jan et al. 2021) using the MACS system according to manufacturer’s instructions. Both fractions were subsequently expanded in growth medium until they became subconfluent. At this point, cells were trypsinized and frozen in growth medium supplemented with 10% (v/v) DMSO. Cells were stored in liquid nitrogen until further use. Only the myogenic (CD56+) fraction of cells was used for experiments described in this study. Comparative analysis of functional characteristics of CD56 + and CD56- cells was described in detail (Jan et al. 2021).

Prior to the experiment, myoblasts were seeded either on uncoated plates (protocol 1) or on Matrigel-coated plates (protocol 2). Differentiation of myoblasts into myotubes was initiated by switching to the differentiation medium (Advanced MEM supplemented with 2% (v/v) FBS, 0.3% (v/v) Fungizone, 0.15% (v/v) Gentamicin, GlutaMax, and vitamins). Experiments were performed after 7–10 days of differentiation in serum-free DMEM (1 g/L) without any additives (protocol 1) or in serum-free Advanced MEM supplemented with Glutamax and vitamins (protocol 2).

### Rat L6 skeletal muscle cells

L6 cells were cultured, as described (Dolinar et al. 2018; Pirkmajer et al. 2020; Skorja Milic et al. 2021; Vidovic et al. 2024). Briefly, myoblasts were cultured in MEMα with nucleosides (MEMα+), supplemented with 10% (v/v) FBS, 1% (v/v) PenStrep (50 U/mL penicillin and 50 µg/mL streptomycin), and 0.3% (v/v) Fungizone (0.75 µg/mL amphotericin B). Differentiation of L6 myoblasts into myotubes was initiated by switching to MEMα + with 2% (v/v) FBS, 1% (v/v) PenStrep and 0.3% (v/v) Fungizone. Experiments were performed and completed within 8–10 days of differentiation in MEMα without nucleosides (MEMα-), FBS, and antibiotics/antimycotics. Cells were cultured at 37 °C in humidified air with 5% (v/v) CO_2_. All experiments were performed under normoxic conditions.

### Immunoblotting

At the end of the experiment, the cells were washed 3 times with phosphate-buffered saline (PBS: 137 mM NaCl, 2.7 mM KCl, 10 mM Na_2_HPO_4_, 1.8 mM KH_2_PO_4_, pH 7.4). Cells were lysed in Laemmli buffer (62.5 mM Tris-HCl (pH 6.8), 2% (w/v) sodium dodecyl sulphate (SDS), 10% (w/v) glycerol, 5% (v/v) 2-mercaptoethanol, 0.002% (w/v) bromophenol blue). After sonication, the lysates were heated at 56 °C for 20 min. Samples were loaded onto a precast 4–12% Bis-Tris polyacrylamide gel, resolved, and subsequently transferred to a polyvinylidene difluoride (PDVF) membrane using the Criterion™ system cell and blotter (from Bio-Rad). The proteins on the membranes were stained with Ponceau S (0.1% (w/v) in 5% (v/v) acetic acid) to evaluate the loading of the samples and the efficiency of the transfer. After blocking with 7.5% (w/v) low-fat dry milk in Tris-buffered saline with Tween (TBST: 20 mM Tris, 150 mM NaCl, 0.02% (v/v) Tween 20, pH 7.5) for 1 h at room temperature, the membranes were incubated overnight at 4 °C with primary antibodies. The membranes were then incubated with the horseradish peroxidase-conjugated secondary antibodies in 5% (w/v) low-fat dry milk in TBST for 1 h at room temperature. The immunoreactive bands were detected by the enhanced chemiluminescence (ECL) method using Immobilon Crescendo Western HRP substrate from Millipore (Billerica, Massachusetts, USA). The signal was recorded with Fusion FX (Vilber (Paris, France)).

When removal of antibodies (“stripping”) was needed for detection of another protein in the same region of the PVDF membrane, we incubated the membranes in the stripping buffer (62.5 mM Tris (pH 6.8), 2% (m/v) SDS, 0.7% (v/v) 2-mercaptoethanol) with constant shaking at 50 °C for 1 h. After washing with TBST and blocking in 7.5% (w/v) low-fat dry milk, detection of previously bound primary antibodies was attempted with the secondary antibody, according to the procedure described above. Lack of immunoreactive bands was taken as evidence that primary antibodies had been removed successfully. This verification step was performed at exactly the same conditions, including exposure time for the (potential) detection of immunoreactive bands, as for the next detected target protein. Once successful removal of primary antibodies was verified, the membranes were incubated with a new primary antibody overnight. All subsequent procedures were the same as described above.

### Measurement of protein concentration

Protein concentration in cell lysates was measured using the Pierce 660 nm Protein Assay (Thermo Fisher Scientific, #22,660), a colorimetric assay suitable for samples prepared in Laemmli buffer.

### Quantitative real-time polymerase chain reaction (qPCR)

Cells were lysed with GTC buffer supplemented with 2% (v/v) 2-mercaptoethanol. Total RNA was extracted using the E.Z.N.A. HP Total RNA Isolation Kit and reverse transcribed using the High-Capacity cDNA Reverse Transcription Kit. qPCR was performed on a QuantStudio 3 Real-Time PCR System (Applied Biosystems Thermo Fischer scientific (Waltham, MA, USA) in a 96-well format using TaqMan chemistry and TaqMan gene expression assays. Expression of target genes was normalised using cyclophilin A (PPIA) as an endogenous control. Standard quality controls, such as the use of equal RNA concentrations in reverse transcription (RT) reactions, RT negative controls (i.e. controls, to which all reagents for reverse transcription except reverse transcriptase were added), and non-template controls (NTC, i.e. controls, to which all reagents for PCR except cDNA were added), were done routinely for all PCR analyses in accordance with the MIQE guidelines (Bustin et al. 2009). Expression levels were calculated as gene expression ratios using the following equation:

$$\text { gene expression ratio }=\frac{E_{\text {endogenous control }}^{\text {Ct(endogenous control})} \text {}}{E_{\text {target gene}}^{\text {Ct(target gene})}}$$

with equal threshold signal setup for both genes. Efficiency of the PCR reaction was calculated with the LinRegPCR software (Ramakers et al. 2003; Ruijter et al. 2009).

### Statistical analysis

Data are presented as mean ± standard error of the mean (SEM) and were statistically analysed using Prism version 9.0 (GraphPad Software, San Diego, CA, USA). Statistical differences were assessed with a one-way ANOVA (followed by Dunnett’s or Tukey’s test) or Kruskal-Wallis test (followed by Dunn’s test). When treatments were compared only with the control (Basal), Dunnett’s (for parametric) or Dunn’s test (for nonparametric data) was used. Tukey’s test was used after one-way ANOVA when multiple groups were compared among each other.

## Results

### Effect of MG132 and PYR-41 on the PDK1 expression in cultured primary human myotubes

The proteasome inhibitor MG132 and PDK inhibitor dichloroacetate (DCA) reduced the PDK1 protein levels in rat L6 myotubes (Skorja Milic et al. 2021). Notably, DCA did not affect *Pdk1* mRNA expression, indicating it acted on a posttranscriptional level (Skorja Milic et al. 2021). Important functional differences between in vitro skeletal muscle cell models have been previously noted (Abdelmoez et al. 2019; Pirkmajer et al. 2020; Pirkmajer and Chibalin 2011; Vidovic et al. 2024), which is why the first objective of this study was to evaluate whether this effect is present also in primary human myotubes. Under basal conditions, primary human myotubes expressed all four PDK isozymes, albeit to very different extents (Table 2). As estimated by the gene expression ratios (*PDK* mRNA/*PPIA* mRNA), *PDK2* mRNA predominated, followed by *PDK4*, *PDK3*, and *PDK1* mRNA.

**Table 2:** The mRNA expression levels of PDK isozymes in cultured human myotubes. Gene expression ratio (*PDK*/*PPIA*) was determined in cultured myotubes from 4 human donors. Cyclophilin A (*PPIA*) was used as endogenous control

PDK isozyme	*n*	Gene expression ratio (*PDK*/*PPIA*)	
		Mean	SD
*PDK1*	4	0.00075	0.00018
*PDK2*	4	0.458	0.309
*PDK3*	4	0.035	0.015
*PDK4*	4	0.096	0.077

**Fig. 1:**
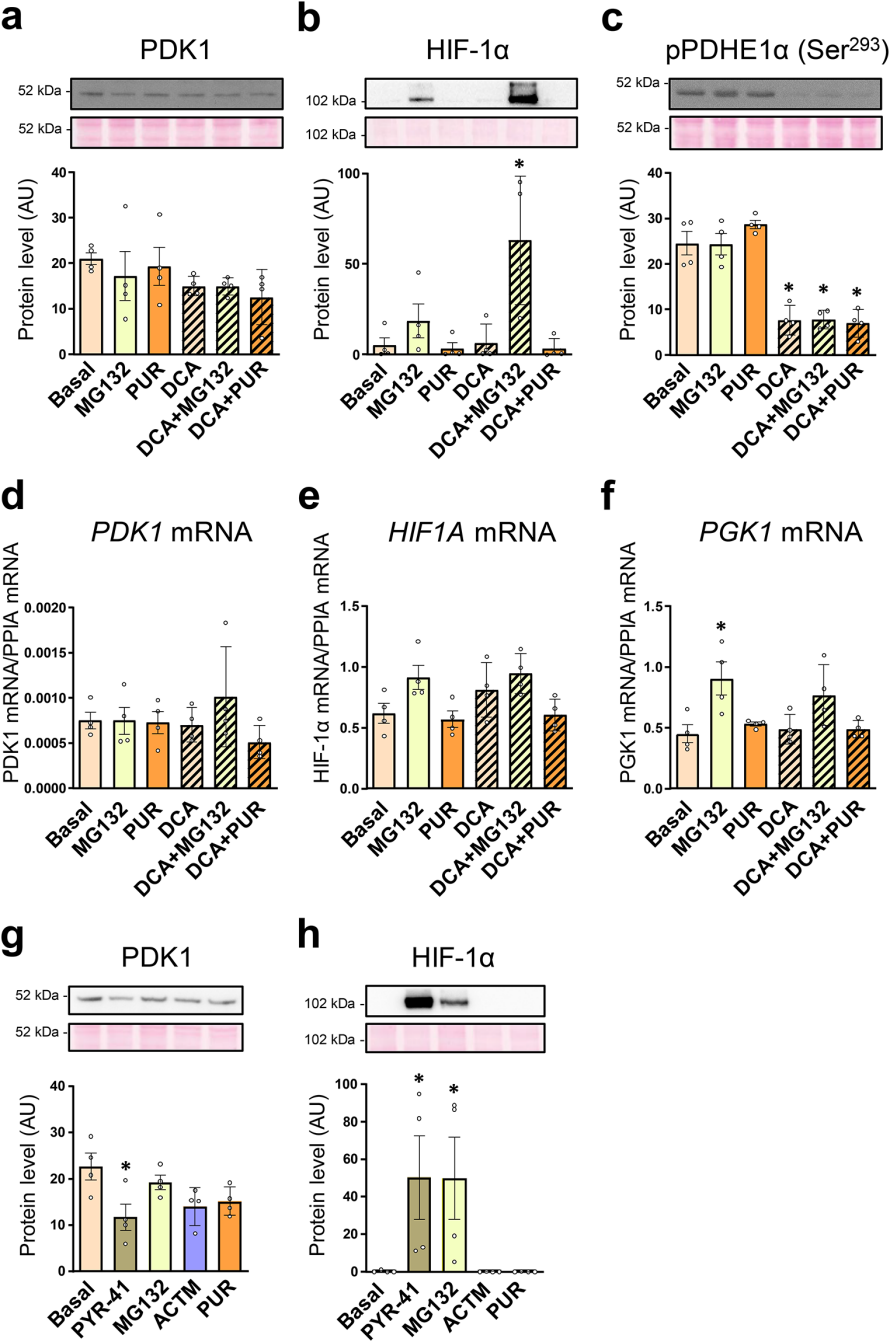
Effect of MG132 and PYR-41 on the PDK1 expression in cultured primary human myotubes. (**a**-**f**) Myotubes, cultured in serum-free DMEM, were treated with MG132 (1 µM), puromycin (0.3 µg/ml, PUR), and/or DCA (10 mM) for 24 h. (**g**,** h**) Myotubes, cultured in serum-free Advanced MEM (containing insulin), were treated with PYR-41 (50 µM), MG132 (1 µM), actinomycin D (5 µg/mL, ACTM), or PUR (0.5 µg/mL) for 24 h. Immunoblotting was used to assess the abundance of (**a**,** g**) PDK1, (**b**,** h**) HIF-1α, (**c**) phosphorylated PDHE1α (Ser293). qPCR was used to estimate the expression of (**d**) *PDK1* mRNA, (**e**) HIF-1α (*HIF1A*) mRNA (**f**) and *PGK1* mRNA (endogenous control: *PPIA* mRNA). Membranes stained with Ponceau S are shown as loading controls. Data are shown as means ± standard error, *n* = 4. *P < 0.05 vs. Basal, One-way ANOVA followed by Dunnett’s test (**a-g**), Kruskal-Wallis test followed by Dunn’s test (**h**)

To assess the turnover of PDK1, primary human myotubes, cultured in serum-free DMEM, were treated with the proteasome inhibitor MG132 (1 µM), the translation inhibitor puromycin (0.3 µg/ml), and/or the PDK inhibitor DCA (10 mM) for 24 h (Fig. 1a-f). Treatment with MG132 did not increase PDK1 protein (Fig. 1a) or mRNA levels (Fig. 1d) despite the induction of HIF-1α (Fig. 1b, e). In contrast, phosphoglycerate kinase 1 (Fig. 1f), which is known to be regulated by HIF-1α in primary human myoblasts (Pirkmajer et al. 2010), was upregulated by MG132. Inhibition of translation by puromycin did not result in a significant reduction in PDK1 protein levels, suggesting that PDK1 has a relatively long half-life in these cells. In contrast to what we had previously observed in L6 myotubes (Skorja Milic et al. 2021), DCA did not significantly affect PDK1 protein levels (Fig. 1a), but it significantly reduced the abundance of phosphorylated PDHE1α (Ser293) (Fig. 1c), which demonstrates that total PDK activity was effectively suppressed by DCA.

Medium composition is an important source of variability and can significantly modify experimental results (Dolinar et al. 2018; Pirkmajer and Chibalin 2011; Rajh et al. 2016). Since DMEM lacks insulin and other proteins, which could affect protein turnover, including turnover of PDK1, in primary human myotubes, experiments were conducted also using Advanced MEM, which contains insulin, albumin, and transferrin. Primary human myotubes, cultured in Advanced MEM, were treated with the ubiquitination inhibitor PYR-41 (50 µM), MG132 (1 µM), the transcription inhibitor actinomycin D (5 µg/ml), and puromycin (0.5 µg/ml) for 24 h (Fig. 1g, h). PYR-41 and MG132 did not increase PDK1 protein levels (Fig. 1g), although HIF-1α was prominently upregulated (Fig. 1h). Since ubiquitination directs HIF-1α to proteasomal degradation, this result indicated that PYR-41 and MG132 effectively inhibited ubiquitination and the proteasome, respectively. Puromycin and actinomycin did not have a significant effect on the abundance of PDK1, which again suggested that PDK1 has a relatively long half-life and slow turnover in these cells.

### Effect of chloroquine and MG132 on the expression of PDK1 in L6 myotubes

Failure of MG132 to increase PDK1 levels in cultured human myotubes was broadly compatible with results that we had previously obtained in rat L6 myotubes (Skorja Milic et al. 2021). However, in L6 myotubes MG132 had not only failed to increase the abundance of PDK1 but had actually decreased it (Skorja Milic et al. 2021). We therefore focused our attention on the effect of MG132 on PDK1 in L6 myotubes.

**Fig. 2:**
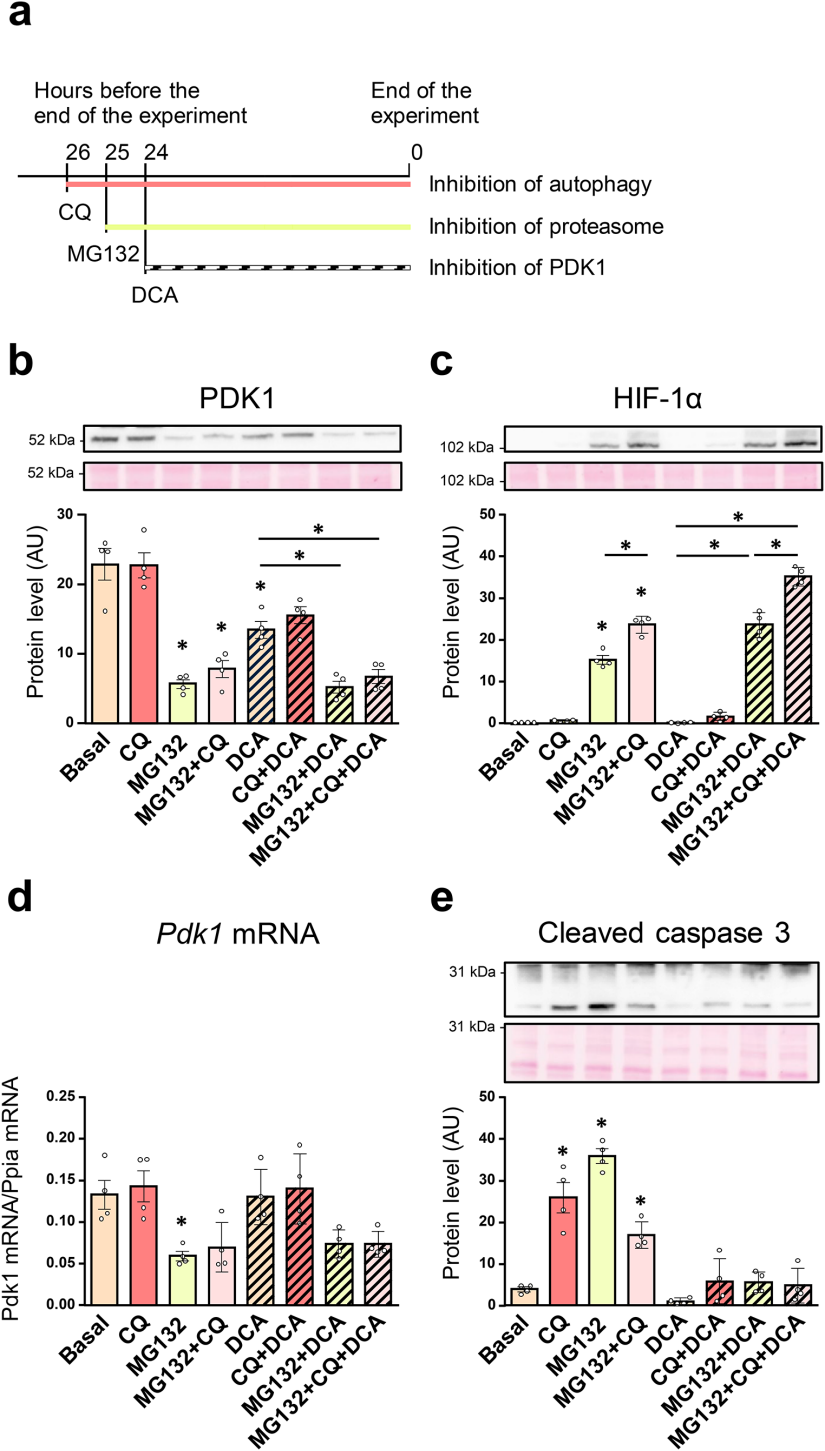
Effect of chloroquine and MG132 on the expression of PDK1 in L6 myotubes. (**a-e**) L6 myotubes, cultured in serum-free MEMα, were treated with vehicle (0.1% DMSO), chloroquine (10 µM for 26 h, CQ), MG132 (1 µM for 25 h), and/or DCA (10 mM for 24 h). (**a**) Schematic overview of the experiment. Immunoblotting was used to assess the abundance of (**b**) PDK1, (**c**) HIF-1α, and (**e**) cleaved caspase 3. qPCR was used to estimate the expression of (**d**) *Pdk1* mRNA (endogenous control: *Ppia* mRNA). Data are shown as means ± standard error, *n* = 4. *P < 0.05 vs. Basal or as indicated (One-way ANOVA followed by Tukey’s test)

MG132 can direct ubiquitinated proteins to autophagic degradation (Wang et al. 2019), which could explain the reduction of PDK1 levels during the MG132 treatment in L6 myotubes. Autophagy in L6 cells was effectively inhibited by 5 µM chloroquine (Dong et al. 2019). To determine whether autophagy is involved in PDK1 degradation during proteasome inhibition, L6 myotubes were treated with chloroquine (10 µM, 26 h), MG132 (1 µM, 25 h), and/or DCA (10 mM, 24 h) (Fig. 2a). L6 myotubes were pretreated with chloroquine and MG132 prior to the addition of DCA to inhibit the proteasome and autophagy before PDK1 was inhibited with DCA (see schematic overview in Fig. 2a). PDK1 protein levels were decreased by MG132 and DCA, consistent with our previous study (Skorja Milic et al. 2021), while chloroquine had no effect (Fig. 2b). HIF-1α, a transcriptional regulator of PDK1, was markedly upregulated by MG132 (Fig. 2c), indicating efficient inhibition of the proteasome. Despite the upregulation of HIF-1α, the expression of *PDK1* mRNA (Fig. 2d) was suppressed by MG132. As estimated by measuring the level of cleaved caspase 3, MG132 and chloroquine activated caspase 3 in the absence but not in the presence of DCA (Fig. 2e). Taken together, these results indicate that inhibition of the proteasome does not lead to autophagic degradation of PDK1.

### Effect of PYR-41 and high concentration of chloroquine on the expression of PDK1 in L6 myotubes

**Fig. 3:**
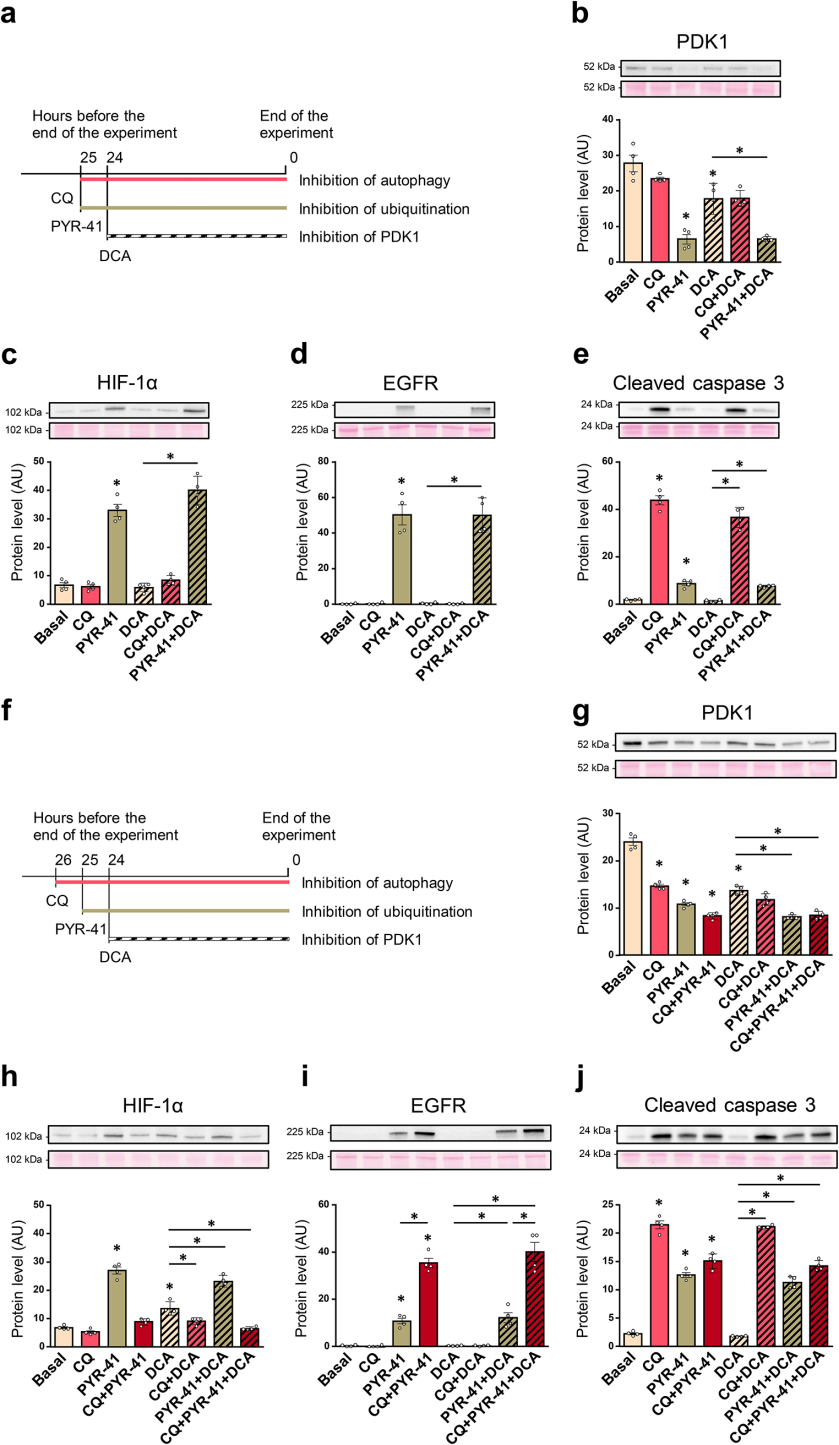
Effect of PYR-41 and high concentration of chloroquine on the expression of PDK1 in L6 myotubes. (**a-e**) L6 myotubes, cultured in serum-free MEMα, were treated with vehicle (0.1% DMSO), chloroquine (100 µM for 25 h, CQ), PYR-41 (50 µM for 25 h), and/or DCA (10 mM for 24 h). (**a**) Schematic overview of the experiment. (**f-j**) L6 myotubes, cultured in serum-free MEMα, were treated with CQ (100 µM for 26 h), PYR-41 (50 µM for 25 h), and/or DCA (10 mM for 24 h). (**f**) Schematic overview of the experiment. Immunoblotting was used to assess the abundance of (**b**,** g**) PDK1, (**c**,** h**) HIF-1α, (**d**,** i**) EGFR, and (**e**,** j**) cleaved caspase 3. Data are shown as means ± standard error, *n* = 4. *P < 0.05 vs. Basal or as indicated (One-way ANOVA followed by Tukey’s test)

To further investigate the role of ubiquitination and autophagy in PDK1 turnover, L6 myotubes were treated with PYR-41 (50 µM, 25 h), a high concentration of chloroquine (100 µM, 25 h), and/or DCA (10 mM, 24 h) (Fig. 3a). PYR-41 reduced PDK1 protein levels in the absence or presence of DCA (Fig. 3b). In contrast, protein levels of HIF-1α (Fig. 3c) and the epidermal growth factor receptor (EGFR) (Fig. 3d), which is targeted for lysosomal degradation by ubiquitination (Galcheva-Gargova et al. 1995; Levkowitz et al. 1998; Tomas et al. 2014), were significantly increased by PYR-41, indicating that ubiquitination in L6 myotubes was effectively suppressed by PYR-41. The amount of cleaved caspase 3 was increased by PYR-41 (Fig. 3e), although the increase was markedly lower compared with the chloroquine treatment, indicating PYR-41 was less toxic than high concentrations of chloroquine. High concentrations of chloroquine tended to suppress PDK1 and did not prevent the DCA-mediated reduction in PDK1 levels (Fig. 3b). Chloroquine had no effect on HIF-1α (Fig. 3c) and EGFR levels (Fig. 3d), but increased the abundance of cleaved caspase 3 (i.e. activated caspase 3) (Fig. 3e).

To determine whether inhibition of the ubiquitin-proteasome system by MG132 redirects PDK1 to autophagic degradation, L6 myotubes were simultaneously treated with chloroquine (100 µM, 26 h), PYR-41 (50 µM, 25 h), and/or DCA (10 mM, 24 h) (Fig. 3f). Chloroquine was added 1 h before PYR-41 and 2 h before DCA to achieve sequential inhibition of autophagy and ubiquitination prior to inhibition of PDK1 with DCA. PDK1 protein levels were reduced by all treatments (Fig. 3g). HIF-1α was increased by PYR-41, which was reversed in the presence of chloroquine (Fig. 3h). In contrast, chloroquine enhanced the upregulation of EGFR by PYR-41 (Fig. 3i). Caspase 3 was activated by all treatments except DCA (Fig. 3j).

**Fig. 4:**
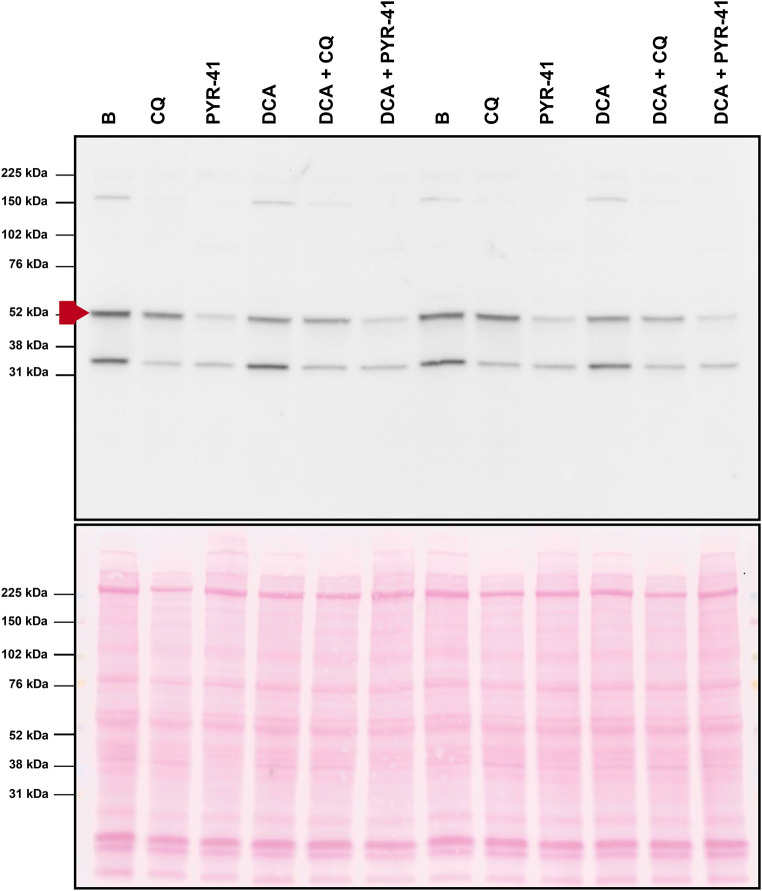
Assessment of the presence of potential proteolytic fragments of PDK1. The band labelled with a red arrow corresponds to PDK1. Two additional bands are observed at 150 kDa and between the 38 and 31 kDa weight markers. Membrane stained with Ponceau S is shown as a loading control. Samples are from the experiment described in Fig. 3a-e

To explore the possibility that DCA, PYR-41, or chloroquine led to cleavage of the PDK1 protein, which could explain the loss of signal at the expected molecular weight, the entire membrane was incubated with the anti-PDK1 antibody (Fig. 4). In addition to the PDK1 band at the ~ 52 kDa marker, two other immunoreactive bands appeared. The ~ 150 kDa band was detectable under basal conditions and in the presence of DCA, but not after treatment with PYR or chloroquine. The second additional band, which had an apparent molecular weight between 31 and 38 kDa, was detectable in all samples and was suppressed by PYR-41 and chloroquine. However, this band did not respond to gene silencing of PDK1 in our previous study (Skorja Milic et al. 2021) in which we validated the anti-PDK1 antibody, indicating that it is most likely not a fragment of PDK1. There were no bands whose intensity would increase in response to treatment with DCA, PYR-41, or chloroquine.

### Effect of actinomycin D and puromycin on the PDK1 protein levels in L6 myotubes

**Fig. 5:**
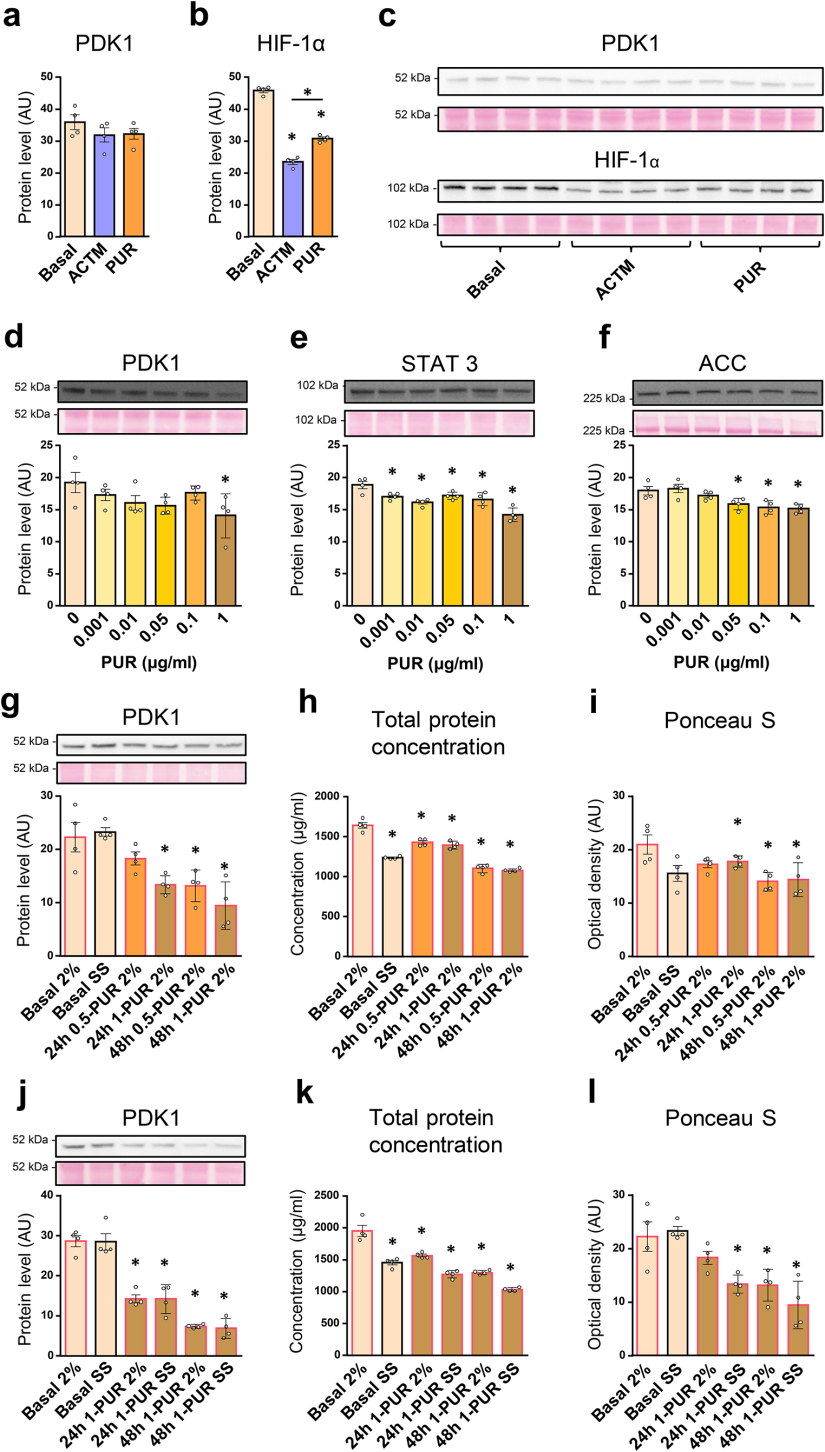
Effect of actinomycin D and puromycin on the PDK1 protein levels in L6 myotubes. (**a**,** b**) L6 myotubes, cultured in serum-free MEMα, were treated with vehicle (0.1% DMSO), actinomycin D (5 µg/mL, ACTM), or puromycin (0.5 µg/mL, PUR). (**d-f**) L6 myotubes, cultured in serum-free MEMα, were treated with puromycin (0.001–1 µg/ml, PUR) for 24 h. (**g-i**)(**j-l**) L6 myotubes were treated with puromycin (0.5 µg/ml, 0.5-PUR or 1 µg/ml, 1-PUR) for up to 48 h. L6 myotubes were grown in MEMα with 2% FBS during the first 24 h of experiment, while they were grown either in the presence (2%) or absence (serum-starved, SS) during the last 24 h of experiment. Immunoblotting was used to assess the abundance of (**a**,** d**,** g**,** j**) PDK1, (**b**) HIF-1α, (**e**) STAT3, and (**f**) acetyl-CoA carboxylase (ACC). Membranes stained with Ponceau S are shown as loading controls. Total protein content was estimated by measuring total protein concentrations in cell lysates (**h**,** k**) and relative optical density of Ponceau S staining of PVDF membranes (**i**,** l**). Data are shown as means ± standard error, *n* = 4. *P < 0.05 vs. Basal (One-way ANOVA followed by Dunnett’s test)

MG132 could theoretically suppress PDK1 by promoting its degradation or by inhibiting its synthesis (Wang et al. 2019). To assess the stability of PDK1 protein, we treated L6 myotubes with actinomycin D (5 µg/ml) and puromycin (0.5 µg/ml) for 12 h (Fig. 5a-c). The protein levels of PDK1 were not changed by these treatments (Fig. 5a, c), while those of HIF-1 were reduced by 40–50% (Fig. 5b, c), which indicated that PDK1 has a longer half-life than HIF-1α. L6 myotubes were therefore treated with a range of puromycin concentrations (0.001–1 µg/ml) for 24 h (Fig. 5d-f), resulting in a significant reduction in protein levels of PDK1 (Fig. 5d). To determine whether unrelated proteins displayed a similar response, we also measured the abundance of STAT3, a transcription factor, and acetyl-CoA carboxylase, a metabolic enzyme that converts acetyl-CoA to malonyl-CoA. Puromycin reduced both the abundance of STAT3 (Fig. 5e) and acetyl-CoA carboxylase (Fig. 5f).

These experiments were performed in a serum-free medium, which may have affected synthesis of PDK1 as serum starvation is known to induce complex responses in cultured cells. Treatment with puromycin was therefore performed also in the presence of serum (2% FBS). To determine the concentration- and time-dependency of the response, L6 myotubes were treated with 0.5 or 1 µM puromycin for 24–48 h in the presence of 2% FBS (Fig. 5g-i). In addition, we did a comparative experiment in which L6 myotubes were treated with 1 µM puromycin in the absence of FBS (serum starvation) and in the presence of 2% FBS (Fig. 5j-l). PDK1 levels were similar in the presence or absence of serum (Fig. 5g, j). Puromycin had a clear time-dependent effect on PDK1 levels, which was not modified by the presence of 2% FBS, indicating expression of PDK1 is not driven by serum components. As estimated by measuring the total protein concentrations in lysates of myotubes (Fig. 5h, k) and optical density of Ponceau S staining of PVDF membranes (Fig. 5i, l), puromycin and serum starvation synergistically suppressed the total protein content in cultured myotubes. The pattern of PDK1 and total protein response was similar, but the suppression of PDK1 levels during puromycin treatment was more pronounced than the suppression of total proteins. The reduction of PDK1 levels during the puromycin treatment indirectly suggested that PDK1 is constantly being degraded in L6 myotubes.

## Discussion

In our previous study, DCA reduced the protein level of PDK1 in rat L6 myotubes (Skorja Milic et al. 2021), which could not be prevented by the proteasome inhibitor MG132. Interestingly, MG132 itself reduced the abundance of PDK1 despite significant inhibition of the proteasome (Skorja Milic et al. 2021), suggesting that PDK1 may have been degraded via a different proteolytic pathway. In the present study, MG132 did not increase PDK1 levels in primary human myotubes, whereas PYR-41 decreased them, indicating that PDK1 degradation in both human and rat skeletal muscle cells occurs despite inhibition of the ubiquitination or the proteasome. Chloroquine, an inhibitor of autophagy, did not prevent the suppression of PDK1 by MG132 in L6 myotubes, suggesting that autophagy is not a major pathway of PDK1 degradation during proteasome inhibition. In addition, PYR-41, an inhibitor of ubiquitination, mimicked the action of MG132 in L6 myotubes, further supporting the idea that the ubiquitin-proteasome system is not essential for PDK1 degradation in cultured skeletal muscle cells. Taken together, our results provide novel insights into mechanisms that underlie PDK1 turnover in L6 myotubes. In addition, they provide additional evidence that proteasome inhibitor MG132 can reduce the protein levels of specific proteins such as PDK1.

Expression of PDK1 was previously detected in cultured primary human skeletal muscle cells (Abbot et al. 2005), but without comparative expression analysis. As estimated from the gene expression ratios, our results show that primary human myotubes express PDK isoforms in the following order *PDK2* > *PDK4* (20% of *PDK2*) > *PDK3* (10% of *PDK2*) > *PDK1* (0.2% of *PDK2*), which largely corresponds to their expression pattern in vivo, where *PDK4* accounts for 15–26% and *PDK1* and *PDK3* account for 1% of *PDK2* mRNA levels (Majer et al. 1998; Spriet et al. 2004). Importantly, the low expression of PDK1 does not mean that it has no physiological function. In PDK2 knock-out mice (Dunford et al. 2011), the total PDK activity is significantly reduced despite marked upregulation of PDK1, which is not unexpected as PDK2 is normally much more abundant than PDK1. However, despite the loss of PDK activity, these knock-out mice have lower pyruvate dehydrogenase activity than their wild-type counterparts. Thus, it appears that although the upregulation of PDK1 could not compensate for the loss of PDK2 activity, it was still able to effectively inhibit the pyruvate dehydrogenase complex (Dunford et al. 2011).

In lung cancer cells, MG132 led to autophagic degradation of the anterior gradient protein 2 homologue (AGR2) (Wang et al. 2019). In these cells, chloroquine (10 µM) counteracted the loss of ARG2 caused by MG132. However, in L6 myotubes, chloroquine was unable to reverse the reduction in PDK1 levels during treatment with MG132, even at high concentrations (100 µM). And although DCA may activate autophagy (Lin et al. 2014), chloroquine failed to halt the loss of PDK1 in L6 myotubes during DCA treatment. While one of the limitations of our study is that we did not monitor autophagy, for instance by measuring the abundance of microtubule-associated protein 1 light chain 3 (LC3) or p62, 5 µM chloroquine was previously shown to be sufficient to suppress autophagy in L6 cells (Dong et al. 2019). Since neither 10 nor 100 µM chloroquine increased PDK1 levels, it seems unlikely that PDK1 is directed towards autophagic degradation when L6 myotubes are exposed to MG132 or DCA. Combined treatment with chloroquine and PYR-41, which markedly increased EGFR protein levels, also reduced the abundance of PDK1, which supports the notion that autophagy is likely not involved in degradation of PDK1. Taken together, our results suggest that PDK1 and AGR2 are regulated by different mechanisms or that the mechanisms described for AGR2 in cancer cells do not exist in L6 myotubes.

The loss of proteins during inhibition of the proteasome could not only be a real biological phenomenon, but also an experimental artefact. The ubiquitination of cystic fibrosis transmembrane conductance regulator (CFTR) resulted in an apparent decrease in its levels during inhibition of the proteasome, as its ubiquitinated immature forms were lost in the sediment when samples were prepared in a Tris-based homogenisation buffer without sodium dodecyl sulphate (Ward et al. 1995). To avoid the formation of sediment and the loss of part of the sample, we lysed L6 myotubes directly in Laemmli buffer containing 2% (w/v) sodium dodecyl sulphate. Since whole cell lysates were used for all immunoblots, it is unlikely that loss of ubiquitinated PDK1 during sample preparation could explain our results. Furthermore, if ubiquitinated PDK1 were lost due to altered solubility, inhibition of ubiquitination by PYR-41 should prevent this loss, which is exactly the opposite of what we observed in L6 myotubes.

MG132 induced HIF-1α but did not alter or even decreased *PDK1* mRNA expression in human and L6 myotubes, respectively. The upregulation of PDK1 by HIF-1α under hypoxic conditions is important for the inactivation of the pyruvate dehydrogenase complex, which switches glucose metabolism from oxidation to lactate formation in cultured fibroblasts and cancer cells (Kim et al. 2006; Papandreou et al. 2006). Induction of HIF-1α with dimethyloxalylglycine, which inhibits the hydroxylation and thus the degradation of HIF-1α, induced the expression of *Pdk1* mRNA in C2C12 cells (Lindholm et al. 2014). Simultaneous upregulation of HIF-1α and PDK1 was also observed in skeletal muscle from PDK2 knock-out mice (Dunford et al. 2011) and from mice and rats exposed to hypoxia (De Palma et al. 2007; Le Moine et al. 2011).

**Fig. 6:**
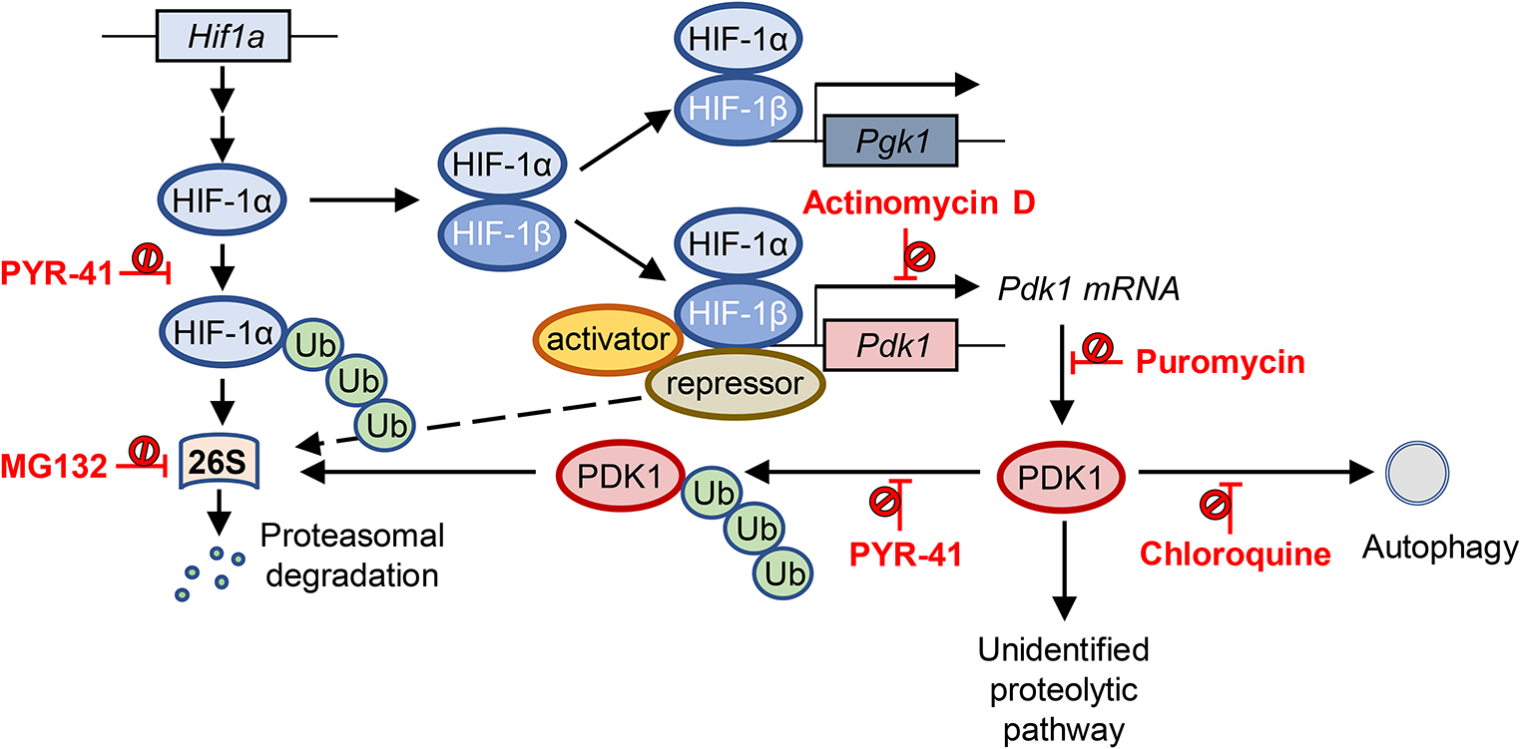
Hypothetical turnover of PDK1 in cultured myotubes with the sites of action of MG132, PYR-41, chloroquine, actinomycin D, and puromycin. Since MG132 and PYR-41 did not upregulate the PDK1 protein, the degradation of PDK1 in myotubes is apparently not suppressed by the inhibition of the ubiquitin-proteasome system. Chloroquine with or without MG132 or PYR-41 also did not increase PDK1 protein levels, indicating that inhibition of autophagy also does not suppress the degradation of PDK1. PDK1 expression is thought to be controlled by HIF-1α, but upregulation of HIF-1α by MG132 or PYR-41 did not result in upregulation of PDK1. This could be explained if inhibition of proteasomal degradation upregulates a putative repressor that blocks HIF-1α action on the *Pdk1* gene. Alternatively, HIF-1α could require the presence of a putative activator whose activity is lost when the proteasome is inhibited

Phosphoglycerate kinase 1 (Fig. 6), which is known to be regulated by HIF-1α in primary human myoblasts (Pirkmajer et al. 2010), was upregulated by MG132 in primary human myotubes. However, our results in the current and previous study (Skorja Milic et al. 2021) show that upregulation of HIF-1α per se does not seem to be sufficient to increase transcription of the *Pdk1* gene in L6 myotubes. In fact, MG132 upregulated HIF-1α, but decreased both PDK1 mRNA and protein levels. Thus, it appears that the upregulation of PDK1 in skeletal muscle cells requires the presence of another transcription factor or co-regulator that is inactivated upon treatment with MG132 or PYR-41. Alternatively, MG132 and PYR-41 could also inhibit the degradation of a repressor that prevents the upregulation of PDK1 by HIF-1α (Fig. 6). While HIF-1α downstream targets, such as PDK1, phosphoglycerate kinase 1 and vascular endothelial growth factor, may show coordinated or divergent responses depending on the stimulus (Lojk et al. 2015; Pirkmajer et al. 2010; Skorja Milic et al. 2021), it needs to be emphasized that a MG132 time course was not performed in the current study. The possibility that upregulation of PDK1 would have occurred at a different time point therefore cannot be excluded with certainty. This explanation is less likely though since our previous experiments with MG132 consistently showed downregulation of PDK1 despite upregulation of HIF-1α (Skorja Milic et al. 2021).

By preventing proteasomal degradation of IκB, MG132 reduces transcriptional activity of NF-κB (Nakanishi and Toi 2005; Arlt et al. 2003). In addition, MG132 reduces the content and transcriptional activity of Forkhead (Fox) M (FoxM) (Bhat et al. 2009). A loss of transcriptional activity when cells are treated with MG132 would therefore not be unexpected. The experiments with puromycin showed that inhibition of translation significantly reduced PDK1 protein levels in L6 myotubes within 24 h, while the effect was even more pronounced after 48 h. The suppression of PDK1 levels by puromycin suggests that inhibition of PDK1 synthesis could contribute to reduction of PDK1 protein levels during treatment with MG132. However, inhibition of PDK1 synthesis can explain the reduction of its abundance only if PDK1 is constantly being degraded. Indeed, if degradation of PDK1 were not taking place, the abundance of PDK1 would stay constant during the puromycin and MG132 treatments. In conjunction with previously published results (Nakanishi and Toi 2005; Arlt et al. 2003 Wang et al. 2019), we therefore hypothesise that inhibition of the proteasome by MG132 suppresses the activity of a factor required for PDK1 expression (Fig. 6). Loss of its activity would reduce PDK1 mRNA and protein synthesis, ultimately leading to a reduction in PDK1 protein levels as PDK1 is continuously degraded despite inhibition of the proteasome and autophagy (Fig. 6). Mitochondrial proteases such as Lon, which is known to degrade PDK4 (Boutagy et al. 2023), may be involved in the degradation pathway, but we did not investigate this question experimentally in the present study. The question therefore remains which proteolytic pathway is responsible for degradation of PDK1 in L6 myotubes.

## Conclusions

1. In primary human myotubes, MG132 did not alter PDK1 protein levels despite markedly increasing the abundance of HIF-1α, implying that the proteasome is not essential for turnover of PDK1 in these cells.
2. In L6 myotubes, chloroquine did not prevent the loss of PDK1 during treatment with MG132, indirectly suggesting that MG132 did not direct PDK1 to autophagic degradation.
3. In L6 myotubes, PYR-41 reduced the abundance of PDK1 in the absence or presence of chloroquine, which mimicked effects of MG132.
4. Since MG132 and PYR-41, which inhibit different steps of proteasomal degradation of proteins, both reduce the abundance of PDK1, the suppression of PDK1 is probably not an off-target effect of MG132 or PYR-41, but is due to inhibition of the ubiquitin-proteasome system.
5. Collectively, our results indicate that cultured myotubes degrade PDK1 via a proteolytic pathway that cannot be inhibited by MG132, PYR-41, and/or chloroquine.

### Electronic supplementary material

Below is the link to the electronic supplementary material.

Supplementary Material 1

## Data Availability

Raw data are provided in the supplement.
